# Weight before and after a diagnosis of breast cancer or ductal carcinoma in situ: a national Australian survey

**DOI:** 10.1186/s12885-020-6566-4

**Published:** 2020-02-20

**Authors:** Carolyn Ee, Adele Elizabeth Cave, Dhevaksha Naidoo, Kellie Bilinski, John Boyages

**Affiliations:** 1 NICM Health Research Institute, Western Sydney University, Locked Bag 1797, Penrith, NSW 2751 Australia; 2 ICON Cancer Centre, Sydney Adventist Hospital, Wahroonga, NSW 2076 Australia

**Keywords:** Breast cancer, DCIS, Overweight, Obesity, Weight gain, Australian women, National survey, Prevalence

## Abstract

**Background:**

Overweight/obesity are strongly implicated in breast cancer development, and weight gain post-diagnosis is associated with greater morbidity and all-cause mortality. The aim of this study was to describe the prevalence of overweight/obesity and the pattern of weight gain after diagnosis of breast cancer amongst Australian women.

**Methods:**

We collected sociodemographic, medical, weight and lifestyle data using an anonymous, self-administered online cross-sectional survey between November 2017 and January 2018 from women with breast cancer living in Australia. The sample consisted mainly of members of the Breast Cancer Network Australia Review and Survey Group.

**Results:**

From 309 responses we obtained complete pre/post diagnosis weight data in 277 women, and calculated pre/post Body Mass Index (BMI) for 270 women. The proportion of women with overweight/obesity rose from 48.5% at diagnosis to 67.4% at time of survey. Most women were Caucasian with stage I-III breast cancer (*n* = 254) or ductal carcinoma in situ (DCIS) (*n* = 33) and mean age was 59.1 years. The majority of women (63.7%) reported they had gained weight after diagnosis with an average increase of 9.07 kg in this group. Of the women who provided complete weight data, half gained 5 kg or more, 17.0% gained > 20 kg, and 60.7% experienced an increase in BMI of >1 kg/m^2.^ Over half of the women rated their concern about weight as high. Of those women who gained weight, more than half reported that this occurred during the first year after diagnosis. Two-thirds (69.1%) of women aged 35–74 years gained, on average, 0.48 kg more weight per year than age-matched controls.

**Conclusions:**

Although the findings from this survey should be interpreted cautiously due to a limited response rate and self-report nature, they suggest that women in Australia gain a considerable amount of weight after a diagnosis of breast cancer/DCIS (in excess of age-matched data for weight gain) and report high levels of concern about their weight. Because weight gain after breast cancer may lead to poorer outcomes, efforts to prevent and manage weight gain must be prioritized and accelerated particularly in the first year after diagnosis.

## Background

Breast cancer is the most common cancer in women worldwide and in Australia [1–3]. There were over 2 million new cases of breast cancer (BC) globally in 2018, with this figure expected to rise to over 3 million by 2040 [3], by which time Australia expects to diagnose more than 25,000 new cases annually [2]. Obesity is a known risk factor for BC, particularly for post-menopausal women [4]. Obesity after menopause (from weight gain during either the premenopausal or postmenopausal years) is directly related to an increased relative risk of BC of 1.11 per 5 kg increase in weight [5].

Obesity at diagnosis is associated with worse BC survival and all-cause mortality rates and may increase the risk of cancer recurrence by 30–40% [1, 6]. Furthermore, weight gain is common after BC diagnosis, and may increase the risk of disease recurrence and mortality and have a negative impact on quality of life [1]. Weight gain after BC diagnosis is thought to be multifactorial and may be related to the use of systemic treatment, younger age at diagnosis, as well as changes in lifestyle [1, 7]. Given the growing population of BC survivors and the link between weight gain and adverse health outcomes, research into weight after BC is of critical importance.

The prevalence of weight gain after BC in Australia has not been adequately quantified. One prospective cohort study conducted in Queensland, in women who had been diagnosed with early breast cancer, described an increase in the proportion of women who were overweight/obese from 57% at diagnosis to 68% over 6 years [8]. However, there is no national data currently available and few population studies exist [9]. The aim of this study was to describe the prevalence of self-reported overweight and obesity before and after a diagnosis of BC or Ductal Carcinoma in Situ (DCIS).

## Methods

### Study design and inclusion criteria

A cross-sectional self-administered anonymous survey was conducted in Australia between November 2017 and January 2018 using Qualtrics**®** online survey software [10]. Any woman living in Australia who self-identified as having BC was eligible to complete the survey. A copy of the Participant Information Sheet was provided electronically via a link on the survey website prior to starting the survey, and women were informed that consent was implied upon commencing the survey. This method of consent was approved by the Human Research Ethics Committee (See below for details). The sample included members of the Breast Cancer Network Australia (BCNA) Review and Survey Group comprising BCNA members who had agreed to receive emails about research studies. Limiting research at BCNA to the Review and Survey group allows researchers to access women who are engaged in the research process, while protecting other BCNA members from frequent research requests. The Review and Survey Group (*n* = 1857) represents approximately 2% of all BCNA members and is one of the largest breast cancer consumer groups available for research in Australia, representing an important source of feedback for the research community.

The survey was emailed to 1835 members on December 5th, 2017 and a reminder email sent January 15th, 2018 (Appendix). A smaller sample (*n* = 26) was also drawn from online communities (women’s health organization social media pages and online breast cancer support groups in Australia) or through word of mouth during November and December 2017. Ethics approval for this study was provided by the Human Research Ethics Committee, Western Sydney University (H12444, October 2017).

### Survey instrument

The survey was developed after reviewing previous literature on weight after BC and was subsequently revised to include feedback from six BCNA representatives and several health researchers. The 60-item survey included questions on the sociodemographic characteristics, medical details such as diagnosis and treatment, lifestyle habits, weight status, and weight management. Details of the survey questions are outlined in the Appendix. In this paper, we report on change in weight from time of diagnosis to time of the survey.

### Weight after diagnosis

Women were asked to self-report their weight in kg at the time of diagnosis, and current weight and height (in meters). Body Mass Index (BMI) was calculated from weight and height as weight/height^2^. The pattern of weight since diagnosis was also assessed as “gained weight overall”, “lost weight overall”, “weight stable” or “weight has fluctuated a great deal”. We devised an unvalidated 11-point Likert scale to evaluate concern about weight (using the question “Please rate how concerned you have been over your weight in the last 12 months”) ranging from 0 (not at all concerned) to 10 (very concerned). We further characterized these data into four categories according to the Likert score: No concern (0), A little concerned (1–3), Somewhat concerned (4–7), Very concerned (8–10). Weight at diagnosis was reported by 90% of total respondents (277 women) and current weight by 95% of respondents (293 women).

### Statistical analysis

IBM SPSS**®** statistics package version 23 [11] and Stata**®** statistical software version 13.11 [12] were used to analyze the data presented in this report. We used descriptive statistics to analyze diagnoses, treatments received, and health provider visits of respondents in percentages. Women who did not self-report their weight were excluded from analyses relating to weight. We calculated the percentage of women who were currently overweight (BMI > 25 and < 30) or obese (BMI > 30) and compared this to the proportion who were overweight/obese at time of diagnosis. Current and pre-cancer weight and BMI were reported as a mean and standard deviation. We calculated the number and percentage of women whose BMI changed from healthy (< 25) to unhealthy (BMI > 25) from diagnosis to time of survey, as well as women who reported an increase of BMI of greater than 1 kg/m^2^. Tests for skewness and kurtosis for weight, BMI at diagnosis, current weight and BMI, and weight gain, indicated that our data had a normal distribution.

We described the self-reported weight gain pattern as percentage of body weight at diagnosis, the proportion of women who gained > 5 kg, and the proportion of women who gained 5–10% and > 10% of body weight. We used a paired t-test to compare weight and BMI at diagnosis and weight and BMI at time of survey, and Fisher’s exact test to explore the association between current BMI classification and weight concern. To test the relationship between weight gain and time since diagnosis (and therefore the hypothesis that weight gain increases with time) we performed a Pearson’s correlation. We also categorized time since diagnosis into 2.5 year blocks and ran a one-way analysis of variance (ANOVA) exploring the relationship between time since diagnosis and weight gain in the following groups of women: women who reported gaining weight overall, and who had self-reported weight gain > 5%. We explored the relationship between amount of weight gain and weight gain concern using the Pearson’s chi-squared test.

We calculated the mean weight gain per year in our sample as total weight gain divided by time since diagnosis in years. We removed one outlier who reported gaining 10.5 kg per year over 2 years and reported rate of weight gain across age groups in five-year brackets (see Fig. 3 and Table 5).

#### Comparing rate of weight gain against normative data

To compare weight gain in our sample against normative data in the Australian population, we used data the AusDiab study. The AusDiab study is a large national, longitudinal population-based study involving > 11,000 adults aged 25 years and older. Baseline data collection for the AusDiab study occurred during 1999–2000, with a subsequent 5-year follow-up (during 2004–2005) [13]. The AusDiab study reported the following mean weight gains per year at 5-year follow-up (2004–5): 700 g per year for 25-34yo, 500 g for 35-44yo, 380 g for 45-54yo, 140 g for 55-64yo and 0 g for 65-74yo). For each respondent in our study for which we could calculate a yearly weight gain, we compared this weight gain with the mean weight gain from AusDiab corresponding to the age group of the respondent by subtracting the AusDiab weight gain from the weight gain reported by that respondent in our study. We used Pearson’s chi-squared testing to compare the numbers of women who gained in excess of the rates reported in the AusDiab study across the age groups described above.

## Results

### Survey response

Of the 1857 BCNA members, 283 (15%) responded to the survey. A further 26 women responded to the survey from other channels giving a total of 309 responses.

### Sample characteristics

Demographic characteristics of respondents are described in Table 1. The majority of women were Caucasian (92.5%, *n* = 285) with a mean age of 59.1 years (*SD* = 9.5, range 33–78, *n* = 298). Characteristics were similar across BCNA members and non-BCNA respondents with no differences between these groups on Pearson’s Chi-squared test. The majority of women were either premenopausal (43%) or perimenopausal (12%) at the time of diagnosis. Of the 145 women who were still menstruating at time of diagnosis, 68% were premenopausal and became postmenopausal, 18% were perimenopausal and became postmenopausal, while a smaller number (13%) remained perimenopausal.

**Table 1:** Demographic characteristics of survey respondents

Description	N (responses)	%
State (*n* = 309)		
Australian Capital Territory	14	4.5%
New South Wales	91	29.5%
Northern Territory	0	0.0%
Queensland	48	15.5%
South Australia	28	9.1%
Tasmania	< 5	1.0%
Victoria	95	30.7%
Western Australia	30	9.7%
Education (*n* = 307)		
High school- year 10	30	9.8%
High school- year 12	35	11.4%
Vocational College	55	17.9%
Bachelor’s degree	90	29.3%
Postgraduate degree	97	31.6%
Ethnicity (*n* = 308)		
European/Anglo Saxon/Caucasian	285	92.5%
Asian	5	1.6%
Oceanic (incl. Australian and New Zealand first peoples, Polynesian and Micronesian)	13	4.2%
North/South/Central American	< 5	0.7%
Mixed ethnicity	< 5	0.7%
Indian	< 5	0.3%
Employment (*n* = 308)		
Employee	140	45.5%
Self-employed	33	10.7%
Home duties/caring for children or family	15	4.9%
In education (going to school, university, etc.)	< 5	1.3%
Doing voluntary work	10	3.3%
Unable to work because of illness	6	2.0%
Unable to work for other reasons	< 5	0.3%
Retired	99	32.1%
Relationship Status (*n* = 309)		
Single	39	12.6%
Married/de facto (living with partner)	230	74.4%
In a relationship (not living with partner)	7	2.3%
Divorced/separated	24	7.8%
Widowed	9	2.9%

### Clinical characteristics

#### Diagnoses

Clinical diagnoses of the respondents are summarized in Table 2. The majority of women (82%, *n* = 252) had been diagnosed with non-metastatic BC. The mean time since diagnosis of BC was 8.2 years (SD 5.12, range 1–32 years) and mean age at diagnosis was 50.9 years (SD = 9.02, range 29–74).

**Table 2:** Diagnoses and treatments received

Description	N	%	Missing n (%)
Diagnoses			1 (0.3%)
Ductal Carcinoma In Situ (DCIS)	33	10.7%	
Localised breast cancer	252	81.8%	
Metastatic breast cancer	14	4.6%	
Inflammatory breast cancer	<5	0.7%	
Other including second primary	7	2.3%	
Treatment to the Breast			2 (0.6%)
Lumpectomy alone	<5	0.7%	
Lumpectomy and radiation	129	42.0%	
Mastectomy alone	74	24.1%	
Mastectomy and radiation	71	23.1%	
Lumpectomy and mastectomy alone	10	3.3%	
Lumpectomy, mastectomy and radiation	16	5.2%	
Double mastectomy	5	1.6%	
Reconstruction after mastectomy (*n* = 164)			
No	84	51.2%	
Immediate	37	22.6%	
Delayed	43	26.2%	
Treatment to the Axilla (*n* = 167)			13 (7.8%)
Sentinel node biopsy only	24	15.6%	
Axillary dissection +/− Sentinel node biopsy	56	36.4%	
Axillary dissection +/− Sentinel node biopsy + radiation	72	46.8%	
Radiation only	<5	1.3%	
Intravenous Systemic Therapy			
Chemotherapy without Herceptin	164	53.1%	
Herceptin only	< 5	0.7%	
Chemotherapy + Herceptin	46	14.9%	
None/not reported	97	31.4%	
Hormonal Treatments			
Tamoxifen alone	58	18.8%	
Other	146	47.3%	
None	105	34.0%	
Current use of hormone therapy			
Yes	125	40.5%	

#### Treatments

Women reported receiving a range of BC treatments including surgery and/or radiation, and axillary, systemic and hormonal treatments, which are detailed in Table 2. The most commonly visited health care providers, within the last 12 months, were breast surgeons (*n* = 172), physiotherapists (*n* = 124) and medical oncologists (for chemotherapy) (*n* = 119). On average, respondents (*n* = 247) had visited three health care providers in the last 12 months (range, 1–10). For women with DCIS, 18 (53%) had a mastectomy, 17 (50%) had received radiation and 19 (56%) had received hormonal treatment.

### Weight change

**Fig. 1:**
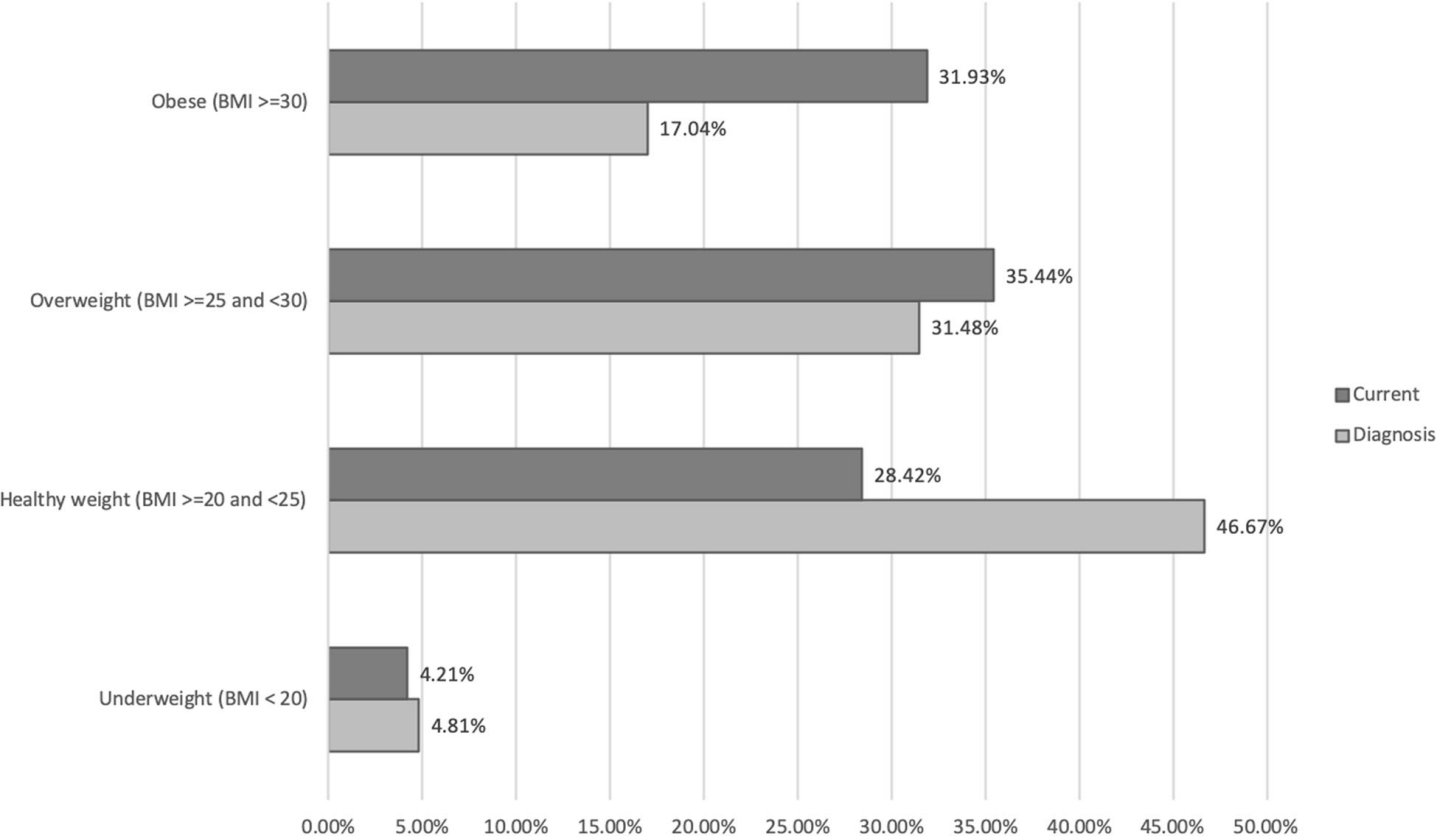
Change in BMI classification after diagnosis of breast cancer. BMI=Body Mass Index

Table 3 and Fig. 1 describe weight and BMI change patterns in our respondents. Mean self-reported weight at time of diagnosis was 71.24 kg (SD 14.01, range 47–158, *n* = 277) and at time of survey was 76.08 kg (SD 15.37, range 46–150, *n* = 293). Mean self-reported current BMI was 28.02 kg/m^2^ (SD = 5.88, *n* = 285) and mean pre-cancer BMI was 26.37 kg/m^2^ (SD = 5.92, *n* = 271). Just under half of women (48.5%) were overweight or obese at time of diagnosis, but by the time of the survey this proportion had risen to 67.3%. This increase was most marked for women who were obese, from 17.0% at diagnosis to 31.9% at the time of the survey. Mean weight gain was 4.50 kg (SD 8.90, *n* = 277).

**Table 3:** Weight change patterns after diagnosis of breast cancer

	Description	N	%	Missing
				N (%)
Self-reported weight gain pattern				17 (5.5%)
	Weight gain	186	63.7%	
	Weight loss	38	13.0%	
	Stable	48	16.4%	
	Fluctuated	20	6.9%	
Calculated % weight change from baseline				32 (10.4%)
	Weight loss	62	22.4%	
	<5% weight gain	53	19.1%	
	5–10% weight gain	64	23.1%	
	>10% weight gain	98	35.4%	
Calculated weight change				32 (10.4%)
	Weight loss	62	22.4%	
	Weight gain up to 5 kg	75	27.1%	
	Weight gain ≥5 kg and < 10 kg	75	27.1%	
	Weight gain ≥10 kg and < 20 kg	18	6.5%	
	Weight gain >20 kg	47	17.0%	
Timing of weight gain^a^ (*n* = 186)				
	<6 months post diagnosis	47	25.3%	
	6–12 months post diagnosis	60	32.3%	
	12–18 months post diagnosis	38	20.4%	
	18–24 months post diagnosis	16	8.6%	
	2–3 years post diagnosis	14	7.5%	
	>3 years post diagnosis	11	5.9%	
Change in BMI classification from time of diagnosis to time of survey (*n* = 270)				
Weight gain	Healthy to overweight	49	18.2%	
	Overweight to obese	40	14.8%	
	Healthy to obese	5	1.9%	
	Underweight to healthy weight	<5	1.5%	
	Underweight to overweight	<5	0.4%	
Stable	Remained in healthy range	69	25.6%	
	Remained in overweight range	39	14.4%	
	Remained in obese range	40	14.8%	
	Remained in underweight range	8	3.0%	
Weight loss	Healthy weight to underweight	3	1.1%	
	Obese or overweight to healthy weight	6	2.2%	
	Overweight to underweight	<5	0.4%	
	Obese to overweight	5	1.9%	

^a^this question was answered only if respondents had selected “gained weight overall”; BMI=Body Mass Index; Underweight = BMI < 20; Healthy weight = BMI > 20 and < 25; Overweight = BMI > 25 and < 30; Obese = BMI > 30

One fifth (54/270, 20.0%) of women went from being in the healthy weight range at diagnosis (BMI <25), to an unhealthy weight range (BMI > 25), a further 14.8% moved from the overweight range into obesity, and 60.7% (164/270) of women reported an increase in BMI greater than 1 kg/m2 (Table 3). Of note, a small proportion of women lost weight whereby 5.6% of women went down at least one BMI category (Table 3).

There was a statistically significant difference between both weight and BMI at diagnosis and current weight and BMI (mean difference 4.50 kg, CI 3.45–5.55, *p =* 0.00, *n* = 277 and 1.64 kg/m^2^, CI 1.24–2.04, *p =* 0.00, *n* = 270 respectively). The majority of respondents (63.7%) reported they had gained weight overall after diagnosis. This is consistent with the self-reported weight gain in our study, where 58.5% of women gained > 5% of pre-diagnosis body weight. Half of the respondents had gained more than 5 kg, with 17.0% reporting gains of over 20 kg of weight.

Of the women who reported gaining weight overall and for whom we had complete weight data (*n* = 175), 87.4% (153/175) gained ≥5 kg of weight, and 54.9% gained > 10% of pre-diagnosis body weight. Average weight gain in this group was 9.07 kg. Women reported that weight gain predominantly occurred within the first 2 years of diagnosis (86.6%) with 57.5% reporting that weight gain mostly occured within the first 12 months. Weight gain was not correlated with time since diagnosis (*n* = 173, *r* = .114, *p* = 0.14). There was no difference in the amount of weight gain by time since diagnosis when this was examined in blocks of 2.5 years, in women who had reported weight gain overall (*n =* 175, *p* = 0.26), and in women who self-reported weight gain of greater than 5% of diagnosis body weight (*n* = 162, *p* = 0.27). (Table 4).

**Table 4:** Weight gain by time in years since diagnosis

	Women who had gained >5% weight (*n* = 162)			Women who reported weight gain pattern overall (*n* = 175)		
	Mean weight gain (kg)	SD	Freq.	Mean weight gain (kg)	SD	Freq.
Time since diagnosis (years)						
< 2.5	9.00	(6.51)	6	8.14	(6.36)	7
2.5–5	8.42	(4.57)	23	7.36	(4.73)	28
5–7.5	9.27	(5.27)	54	8.90	(5.47)	56
7.5–10	9.15	(5.42)	22	8.43	(5.64)	24
> 10	11.21	(7.14)	57	10.38	(7.37)	60

*SD* Standard Deviation, *Freq* Frequency

**Fig. 2:**
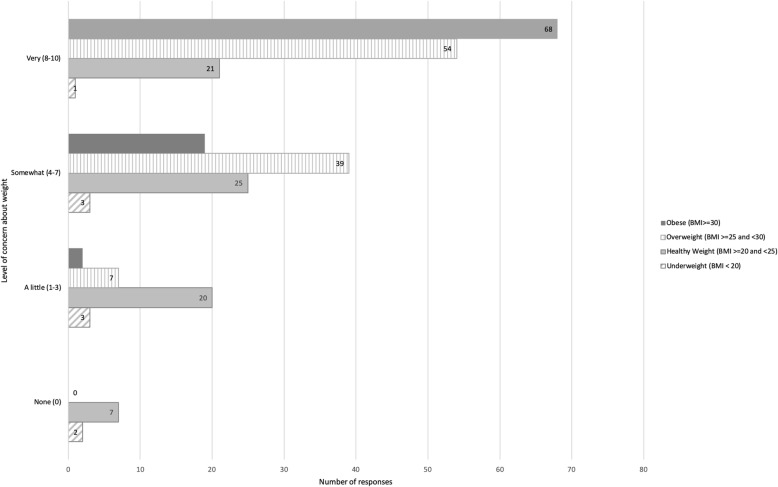
Weight gain concern and current BMI classification (*n* = 285). BMI=Body Mass Index

Three quarters (74.7%, *n =* 68/91) of women who were currently obese reported very high levels of concern about their weight, compared to a quarter of women in the healthy weight range (25.9%, *n =* 21/81) (*p* = 0.00). Women who had gained more weight were more likely to express high levels of concern about their weight. Of the women who gained 5–10% of weight and > 10% of weight, 54.8 and 78.4% reported being very concerned about their weight respectively, compared with 22.5% of women who had gained less than 5% of their diagnosis weight (*X*^2^, (9, *n* = 263) =67.6137, *p* = 0.000). (Fig. 2).

### Rate of weight gain, and comparison with normative data

**Fig. 3:**
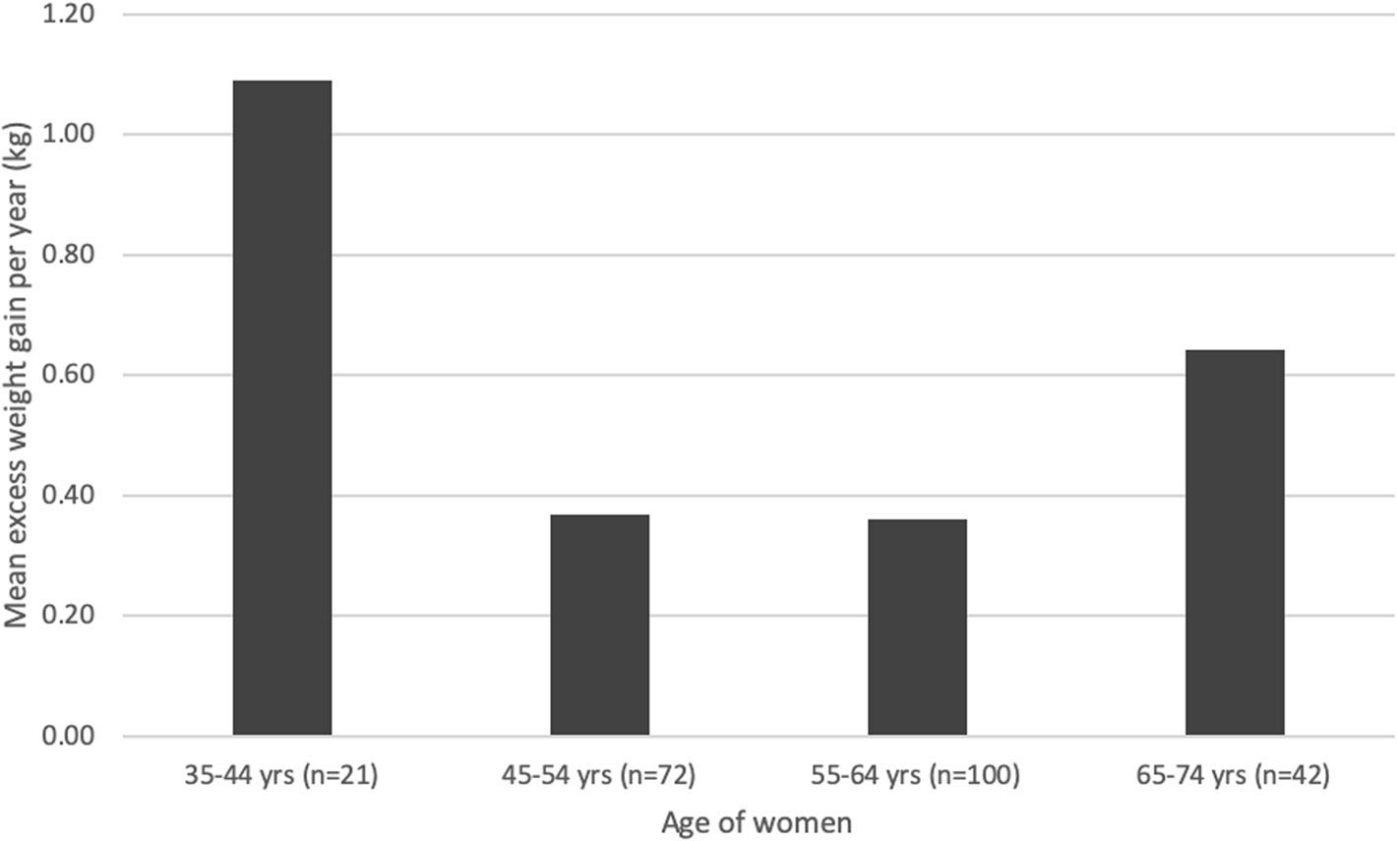
Mean weight gain per year in excess of normative data, by age (*n* = 234)

On average, women in our study gained 0.64 kg per year (*n* = 270, SD = 1.76, range − 8 to 10.5) (see Table 5). For women aged 25–74 years (the age range for which we have normative data), the mean weight gain in excess of age-matched controls was 0.48 kg per year (*n* = 235, SD = 1.67, range − 8.38 to 7.62). Overall, two thirds (69.8%) of women in our sample gained in excess of normative weight gain in the AusDiab study, including 25.1% of women who gained > 1 kg per year in excess of normative rates of weight gain. There was no difference between age groups with regard to the number of women who gained in excess of normative weight gain (*X2,* (*n* = 235) = 6.6929, *p =* 0.153). See Fig. 3 for mean weight gain in excess of normative data for each age group. There was only one woman in the 25–34 age group; to protect confidentiality we did not include her data in Table 5 or Fig. 3.

**Table 5:** Mean weight gain per year in each age group, and proportion who gained in excessive of normative rates

Age (years)	Mean weight gain per year in kg in our study (SD)	% who gained in excess of AusDiab data
35–44 (*n* = 21)	1.59 (1.16)	81.0
45–54 (*n* = 72)	0.75 (2.35)	61.1
55–64 (*n* = 100)	0.50 (1.47)	76.0
65–74 (*n* = 42)	0.39 (0.94)	64.3
All (*n* = 235)	0.64 (1.76)	69.8

## Discussion

This is the first national survey conducted in Australia to describe weight after breast cancer. The distribution of responses according to state and territory in our survey is broadly consistent with the incidence of BC in these regions [14] indicating our sample was nationally representative by location. We found that two-thirds of our respondents were currently overweight or obese, with the majority of women reporting they had gained weight after diagnosis, mostly within the first 12 months and at a substantial self-reported average of 9.07 kg. Of note, the proportion of women who were overweight or obese rose sharply from 48% at time of diagnosis to 67% at the time of the survey, with the proportion of women who were obese almost doubling from 17 to 32%. The majority of women gained weight in excess of the rates reported in age-matched controls without breast cancer. This equated to an average of an additional 2.42 kg over 5 years. A very small proportion of women (5.6%) changed from a higher to lower BMI category. It would be of interest to explore such findings to enquire whether this is a result of intended weight loss or treatment related effects.

The proportion of women who were overweight or obese in our study is consistent with those from a prospective study of 287 women conducted in Queensland, Australia which compared weight gain after diagnosis of early BC. By 6 years, 68% of women in the cohort were overweight or obese, [8] which is remarkably similar to our findings. Median weight gain for study participants between 6 and 72 months was 0.7 kg, and mean BMI increase was 0.2 kg/m^2^. The authors of the cohort study compared the weight gain in the BC cohort with age-matched controls and reported a significant difference, with only 50% of age-matched controls being overweight or obese. One other population study has been published from Shanghai on obesity and clinical outcomes of 4561 Chinese women [9]. In that study, mean weight gain at 18 months post-diagnosis was 1.7 kg. Mean weight gain in our study was significantly higher at 4.5 kg which could be explained by the longer time since diagnosis in our study. Further, the mean weight gain in women who had gained weight overall in our study is substantially higher than what is reported in the Australian cohort study (9.07 kg vs 5.3 kg) although we note that the mean time since diagnosis in our study is 8.2 years, while the cohort study used a 6-year follow-up. Our study provides additional data on weight gain after BC in Australia, over a wider time frame and location and with a larger sample size, and suggests that the problem of weight gain after BC may be larger than previously anticipated.

A large international review found that 50–96% of early stage BC patients experience weight gain during treatment in the range of 1.7 kg to 5.0 kg in the 18 months following treatment [15]. Of those who gained weight, 27% gained 2 kg to 5 kg and 24% gain 5 kg or more in the 18 months following treatment. This compares to our study where 50.55% reported gaining 5 kg or more mainly in the first 18 months after treatment, again suggesting that weight gain after BC is a greater problem than previously thought.

Our findings are of concern because weight gain pre- and post- BC diagnosis have both been associated with increased morbidity and mortality. Whilst those at heaviest weight at diagnosis appear to carry an increased risk, even those within the healthy weight range at diagnosis face increased risk following weight gain [16]. Data from the Nurse’s Health Study in the USA showed the risk of cancer recurrence was increased by 40% following a mean weight gain of 2.7 kg, and by 53% following mean weight gain of 7.7 kg, [17] with the greatest increased risk in those of healthy weight at diagnosis. Another observational study of 3993 women, each 5 kg gain in weight post-diagnosis was associated with a significant 12% increase in all-cause mortality, 13% increase in BC-specific mortality, and 19% increase in cardiovascular-disease mortality (all *p* < 0.05) after an average 6.3 years follow-up after diagnosis [18]. Extrapolating from these results indicate that approximately half of our cohort face a significant increase in cancer recurrence and mortality due to weight gain > 5 kg, and that efforts to prevent weight gain in women diagnosed with BC need to be accelerated and prioritized.

Our findings indicate high levels of concern about weight, particularly in women who were currently overweight or obese. Weight gain exacerbates the significant body image concerns already faced by BC survivors, has a negative impact on quality of life, and may be a cause of distress if it was unanticipated [19]. We did not explore quality of life or levels of distress in our cohort, but additional research in this area appears to be warranted.

Although the proportion of overweight and obesity in our survey is similar to national data for women aged 45–64 (which ranges from 61 to 69%) [20], the majority of our respondents were from a higher socioeconomic group with 60% having a Bachelor’s degree qualification or higher, and 56% being employed or self-employed. National data indicate rates of overweight and obesity for women in the highest socioeconomic group as low as 48% [20] indicating that the proportion of overweight and obesity in our respondents is higher than would be expected of women with similar demographic characteristics. Finally, when compared to age-matched controls from the AusDiab study, 69.8% of women in our survey gained in excess of normative weight gain, indicating that the weight gain experienced within our sample is unlikely to be explained by weight gain that would normally be experienced as women age and progress through the menopausal transition.

This study also highlights the importance of treatment teams being aware that weight gain, particularly in the first year after treatment, is an important issue, which would benefit from interventions such as diet and exercise. In this study, 186 of 292 patients (63.69%) gained weight, 57% gained within the first 12 months and 77% within 18 months. The timing of weight gain within the first year of treatment has been reported by others [21, 22]. Recently, the Clinical Oncological Society of Australia has strongly advocated for exercise to be embedded as part of standard practice in cancer care and advised all members of the multidisciplinary cancer team to promote physical activity, encourage patients to adhere to exercise guidelines and refer patients to an accredited exercise physiologist or physiotherapist with experience in cancer care [23]. All people with cancer should progress towards and, once achieved, maintain participation in at least 150 min of moderate intensity or 75 min of vigorous-intensity aerobic exercise (e.g. walking, jogging, cycling, swimming) each week; and two to three resistance exercise (i.e. lifting weights) sessions each week involving moderate-to-vigorous-intensity exercises targeting the major muscle groups. In women with breast cancer, there appears to be a window of opportunity within the first 18 months to initiate weight management interventions in order to prevent excessive weight gain.

Strengths of this survey include the higher than expected response rate from the BCNA Review and Survey Group. According to the Research and Evaluation Manager, BCNA (email communication 3 Oct 2017), the typical response rate in this group is 10%, whereas the response to our survey was 15%. However, given that the Review and Survey Group represents only approximately 2% of all BCNA members, the validity of our findings is somewhat limited but important to highlight particularly to clinicians managing patients with breast cancer to ensure they encourage and more importantly “prescribe” an exercise program after cancer treatment.

We achieved a broadly nationally representative sample according to location. The percentage of respondents from each Australian State and Territory is similar to national averages on BC incidence as described by the Australian Institute of Health and Welfare cancer data [14].

Limitations of this survey included its self-report nature. In general, people tend to underestimate their weight and overestimate their height with self-reporting [24]. Social desirability bias and response bias may play a part in this inaccuracy. In our cross-sectional study, it is possible that recall bias led to further underestimation of pre-diagnosis weight, therefore inflating the reported weight gain. Additionally, a small proportion of women chose not to report their weight in this survey (10% for pre-diagnosis weight, and 5% for current weight). However, using self-reported weight and height is simple and readily accessible, and is considered less intrusive than objectively measured weight, therefore allowing us to conduct a nation-wide survey and increase the response rate. The true prevalence of weight gain after BC may be different to that found in our survey as women who had gained weight after BC may have been more likely to respond to our survey compared to women who had not gained weight. Nevertheless, the prevalence reported in our survey is remarkably similar to that in prospective cohort studies, suggesting that our data is robust. There is an urgent need to further understand the predictors of weight gain in women with BC. Further planned analyses from our data will include analysis of the predictors of weight gain in our sample, including use of chemotherapy, hormonal therapy, and menopausal stage at diagnosis.

We acknowledge that the inability to provide matched controls in this survey is a limitation. However, we were able to retrospectively match women by age to controls from the 2005 AusDiab study and found that women gained in excess of normative data, although limitations of our comparison is that we could not locate more recent data on normative rates of weight gain, and the duration of weight gain varied in our sample. Furthermore, that our findings are remarkably similar to a cohort study in the state of Queensland in Australia [8] we believe our findings are a reliable representation of breast cancer survivors. Additionally, it would be of interest to look at change in weight over time and according to menopausal status in matched controls. As such this will be examined in a future manuscript.

We were unable to report on the proportion of fat mass gained relative to muscle mass lost, know as sarcopenia. Sarcopenia is common in many women even without body weight change, with 74% of women increasing total body fat relative to lean muscle, [25] with an increased risk from tamoxifen use [26] and common after reduced activity during chemotherapy. Such changes are associated with the development of comorbidities such as diabetes and cardiovascular disease, thereby influencing long-term survival [27]. Excess adiposity is also associated with poorer prognosis through increases in adipose derived circulating estrogens and via increased circulating levels of insulin, insulin-like growth factor and leptin [28].

Another potential weakness of our study is that the vast majority of survey respondents were Caucasian, thereby limiting the generalizability of our data to women from other ethnicities but provides an important perspective over and above the Shanghai study where patients were less overweight or obese at diagnosis and whose diet differed from a Western diet. Previous research from the United States, has shown when compared with non-Hispanic whites, Hispanic and black women have higher rates of obesity (21.8%, compared with 29.4 and 39.2%, respectively), lower rates of meeting physical activity guidelines (19.0%, compared with 12.5 and 17.5%, respectively), and lower intake of three or more servings of fruit and vegetables per day (27.7%, compared with 19.7 and 21.9%, respectively). Understanding this in the Australian context will be an important component of future research [29].

Additionally, although the response rate from the BCNA Review and Survey Group was higher than what is typically seen, this represented a very small proportion of all BCNA members, limiting the validity of our findings. Notwithstanding such limitations, the demographics in our sample (who were predominantly well-educated and either employed or self-employed) are not inconsistent with national data indicating that the incidence of breast cancer is highest in the areas with highest socioeconomic advantage [30]. Additionally, the demographics of the BCNA respondents and non-BCNA respondents were similar, suggesting that our findings can be extrapolated to other BCNA members.

## Conclusion

This is the first national survey of Australian women to describe weight gain after diagnosis of BC. Survey respondents gained a subtantial amount of weight (mean of 9.07 kg), with a doubling of the proportion of women living with obesity. This is coupled with high rates of concern about weight after breast cancer. Given that weight gain after BC may lead to poorer outcomes, there is a need to prioritize and accelerate efforts to assist women to prevent and manage weight gain after BC, particularly during the first 12 months after diagnosis.

## Data Availability

The datasets used and/or analysed during the current study are available from the corresponding author on reasonable request.

## Appendix

### Specific demographic, medical, menopausal and lymphoedema details requested in the survey

***Demographic characteristics.***

State of residence, highest level of education, ethnicity, employment status, relationship status, current age and age at diagnosis were included to describe the characteristics of women.

***Medical details.***

Women were asked about their diagnosis, treatments received including treatments received to the axilla, the number of lymph nodes removed, whether they had a reconstruction, use of hormonal treatments, menopausal state (at diagnosis and current), presence of other medical conditions and symptoms such as hot flushes and the presence and severity of lymphoedema.

Women were asked to describe the type of breast cancer they were diagnosed with as either “ductal cancer in-situ (DCIS)”, “localised stage breast cancer (where your breast cancer is contained within your breast and/or lymph nodes), “metastatic breast cancer (breast cancer that has spread beyond the breast tissue and lymph nodes to distant parts of the body, such as the bones, liver and lungs; also called advanced, secondary or stage four) “ or “inflammatory breast cancer. “For convenience, inflammatory breast cancer and metastatic breast cancer were then combined and referred to as advanced breast cancer. Women were also asked to indicate the treatments they received such as “Lumpectomy alone”, “Lumpectomy and radiation”, “mastectomy alone”, “mastectomy and radiation”, “removal of lymph nodes”, “chemotherapy”, “hormonal therapy”, “targeted therapy (Herceptin)”, and “other”. As chemotherapy is invariably not provided to women with DCIS, we recoded the diagnosis as “localised” if a woman indicated that she had received chemotherapy.

Menopausal state at the time of diagnosis was assessed as either “Premenopausal (regular periods with no menopausal symptoms such as hot flushes)”, “Perimenopausal/in the menopausal transition (no periods for at least 2 months, plus hot flushes)”, “Postmenopausal (no periods for at least 12 months)” or “Previous surgical menopausal (both ovaries or uterus/womb had been removed).” Participants who indicated they were premenopausal or perimenopausal at the time of diagnosis were asked if they were having periods before breast cancer treatment and to describe what has happened to their periods now; “they have stopped”, “they stopped and then started again”, “they have become more irregular”, “no change” or “other”.

Lymphoedema severity was defined as either “no problem (no noticeable swelling)”, “mild (soft swelling that is not obvious to others and comes and goes)”, “moderate (swelling with occasional hardness in some areas that is obvious to others and is always present)”, “severe (profuse swelling with thickened skin, constant hardness, and a very large, heavy arm that is extremely obvious to others and is always present) as described elsewhere [7].”

***Lifestyle habits.***

Women were asked if they had tried the following specific diets in the previous 12 months: Atkins diet (low carbohydrate), 5:2 diet (eat what you want 5 days a week, send your body into starvation mode for 2 days), Paleolithic diet, Dukan diet (High-protein, low-carb), Vegetarian diet, Vegan diet, Weight Watchers diet, Raw food diet, Ultra low-fat diet, Zon diet, Cambridge diet (very low calories), South Beach diet (low-GI), Other. They were asked if they ate at least the recommended serves of fruit and vegetables a day (2 fruit, five vegetable) with answer options of Yes/No. Self-perceived diet quality was assessed as Excellent/Very Good/Good/Fair/Poor. Smoking was assessed as current cigarrete use (Never smoked/Ex smoker/Recently quit ex smoker (smoked in the last 3 months)/Current smoker) and current smokers were asked to indicate the number of cigarettes they smoked each day. Alcohol intake was assessed as Non drinker/1–7 standard drinks a week/8–14 standard drinks a week/> 14 standard drinks a week) and a guide to standard drink sizes was provided. The validated Weight Self Efficacy Scale (WEL-SF) [3] was used to evaluate how confident women now felt about being able to successfully resist the desire to overeat in eight different situations on an 11-point Likert scale from 0 (not confident at all) to 10 (very confident). We further dichotomised the responses into “Not confident” (0–4) and “Confident” (5–10). Physical activity levels were calculated according to the number of 20-min sessions of less vigorous exercise or more vigorous exercise a week, given a weighting and described in terms of MET (metabolic cost) minutes where MET minutes less than 80 were coded as no physical activity, 80 to 400 as low, 400 to 560 as moderate and more than 560 as high. A value of 4 METs was given to moderate physical activity and 7.5 to vigorous physical activity [26].

*Weight management.*

Experiences with a range of weight loss interventions (Exercise, Diet – various: Intermittent fasting, etc. (please specify), Meal replacements e.g. shakes, Medication, Weight loss supplements/products, Surgery (please specify), Online program e.g. 12 week Body Transformation, Social support, Weight loss program e.g. Jenny Craig, Psychological treatments such as CBT (Cognitive Behavioural Therapy) and the perceived effectiveness of the interventions on was described using a five-point Likert scale from 1 (not at all effective) to 5 (very effective). The responses were further dichotomized into 1 to 2 (not effective) and 3 to 5 (effective). Women were also asked about perceived barriers and facilitators to successful weight loss and weight maintenance, and what they believed should be research priorities in this area.

## Abbreviations

BC: Breast Cancer
BCNA: Breast Cancer Network Australia
BMI: Body Mass Index
DCIS: Ductal Carcinoma In Situ
GP: General Practitioner
